# Risk of Hyperlipidemia in Women with Hysterectomy-A Retrospective Cohort Study in Taiwan

**DOI:** 10.1038/s41598-018-31347-z

**Published:** 2018-08-28

**Authors:** Pei-Chen Li, I.-Ju Tsai, Chung Y. Hsu, Jen-Hung Wang, Shinn-Zong Lin, Dah-Ching Ding, Fung-Chang Sung

**Affiliations:** 1 Department of Obstetrics and Gynecology, Hualien Tzu Chi Hospital, Buddhist Tzu Chi Foundation; Tzu Chi University, Hualien, Taiwan; 2 Management Office for Health Data, China Medical University Hospital, Taichung, Taiwan; 3 College of Medicine, China Medical University, Taichung, Taiwan; 4 Graduate Institute of Clinical Medical Science, China Medical University, Taichung, Taiwan; 5 Department of Research, Hualien Tzu Chi Hospital, Buddhist Tzu Chi Foundation; Tzu Chi University, Hualien, Taiwan; 6 Department of Neurosurgery, Hualien Tzu Chi Hospital, Buddhist Tzu Chi Foundation; Tzu Chi University, Hualien, Taiwan; 7 Institute of Medical Sciences, Tzu Chi University, Hualien, Taiwan; 8 Department of Health Services Administration, China Medical University, Taichung, Taiwan

## Abstract

Hysterectomy has been associated with metabolic change and cardiovascular risk for women after removing the uterus, but inconclusive. This large retrospective cohort study evaluated the hyperlipidemia risk for women with a hysterectomy and/or oophorectomy. From claims data of one million people in the National Health Insurance (NHI) database of Taiwan, we established a cohort consisting of 5887 women newly received a surgery of hysterectomy from 2000–2013, 563 women had a hysterectomy and a oophorectomy, and 556 women had a oophorectomy. From the claims data, 28024 women without any of the surgeries were identified to form the comparison cohort, frequency matched by birth year and surgery year of the women with hysterectomy. By the end of 2013, the incidence of hyperlipidemia was 1.3 times greater in women with a hysterectomy than in comparison women (3.43 vs. 2.65 per 100 person-years), with an adjusted hazard ratio (aHR) of 1.27 (95% CI = 1.19–1.35) for hysterectomy women after controlling for age, oophorectomy, hormone therapy and comorbidities. The incidence of hyperlipidemia increased to 4.93 per 100 person-years in women with both a hysterectomy and an oophorectomy. The relative risk of hyperlipidemia was higher for young women than the elderly women with the surgery. Women with comorbidity of obesity, hypertension or diabetes had a higher incidence of hyperlipidemia. In conclusion, the risk of developing hyperlipidemia could be elevated for women who had a hysterectomy and/or an oophorectomy. Women with hysterectomy should routinely monitor their metabolic status, particularly for young women and those with comorbidity of metabolic symptoms.

## Introduction

Cardiovascular disease (CVD) is a leading cause of death in women, and serum cholesterol is one of the well-known risk factors of CVD^1^. Hyperlipidemia and hypertension increase with age and are more prevalent in women after middle age. Hyperlipidemia refers to an increased level of plasma lipids, including triglycerides, cholesterol, cholesterol esters, phospholipids, and plasma lipoproteins^2^. In addition to genetic caused hyperlipidemia, disorders such as diabetes, alcohol consumption, and some medications may lead to metabolic disorders and the development of acquired hyperlipidemia^3,4^. The National Cholesterol Education Panel’s Adult Treatment Program III released in 2001 set the standard lipid levels, commonly used guideline for clinicians^3^.

Hysterectomy is a surgery to remove the uterus, which is one of common gynecologic surgeries for women in both developed and developing areas^5,6^. Hysterectomy is generally recommended for benign clinical indications, such as uterine leiomyoma, abnormal uterine bleeding and endometriosis, in addition to for reproductive cancers^7^. It has been a concern for the potential of leading to hormone changes after hysterectomy and increasing the risk of CVD and other comorbidities^8–10^. The Norwegian health study found women had an adjusted hazard ratio (aHR) of 1.92 (95% CI: 1.51–2.38) to develop CVD after a hysterectomy^10^.

The effect of hysterectomy on the metabolic status, such as lipid profile, has not been well investigated. We assumed that changes in the hormone level post hysterectomy might change the serum lipid profile. We, therefore, investigated the risk of developing hyperlipidemia for women with hysterectomy, using the Taiwan National Health Insurance (TNHI) Database of one million randomly sampling cohort.

## Materials and Methods

### Data source

The TNHI program covers more than 99% of the entire 23.4 million population of Taiwan. Data of registration files and medical claims for all beneficiaries can be linked through encrypted identification numbers in The National Health Insurance Research Database (NHIRD) (details available at: http://nhird.nhri.org.tw/en/index.htm). A sub-dataset of NHIRD containing 1 million people randomly selected from all beneficiaries was used in this study.

Diseases in the claims data were coded with the International Classification of Diseases, 9^th^ Revision, Clinical Modification (ICD-9-CM). The insurance system required the disease diagnosis with valid supporting clinical findings to prevent a medical fraud. This study was approved by the Research Ethics Committee, China Medical University and Hospital (IRB permits number: CMUH-104-REC2-115). Our research was performed in accordance with relevant guidelines/regulations.

### Study subjects

**Figure 1:**
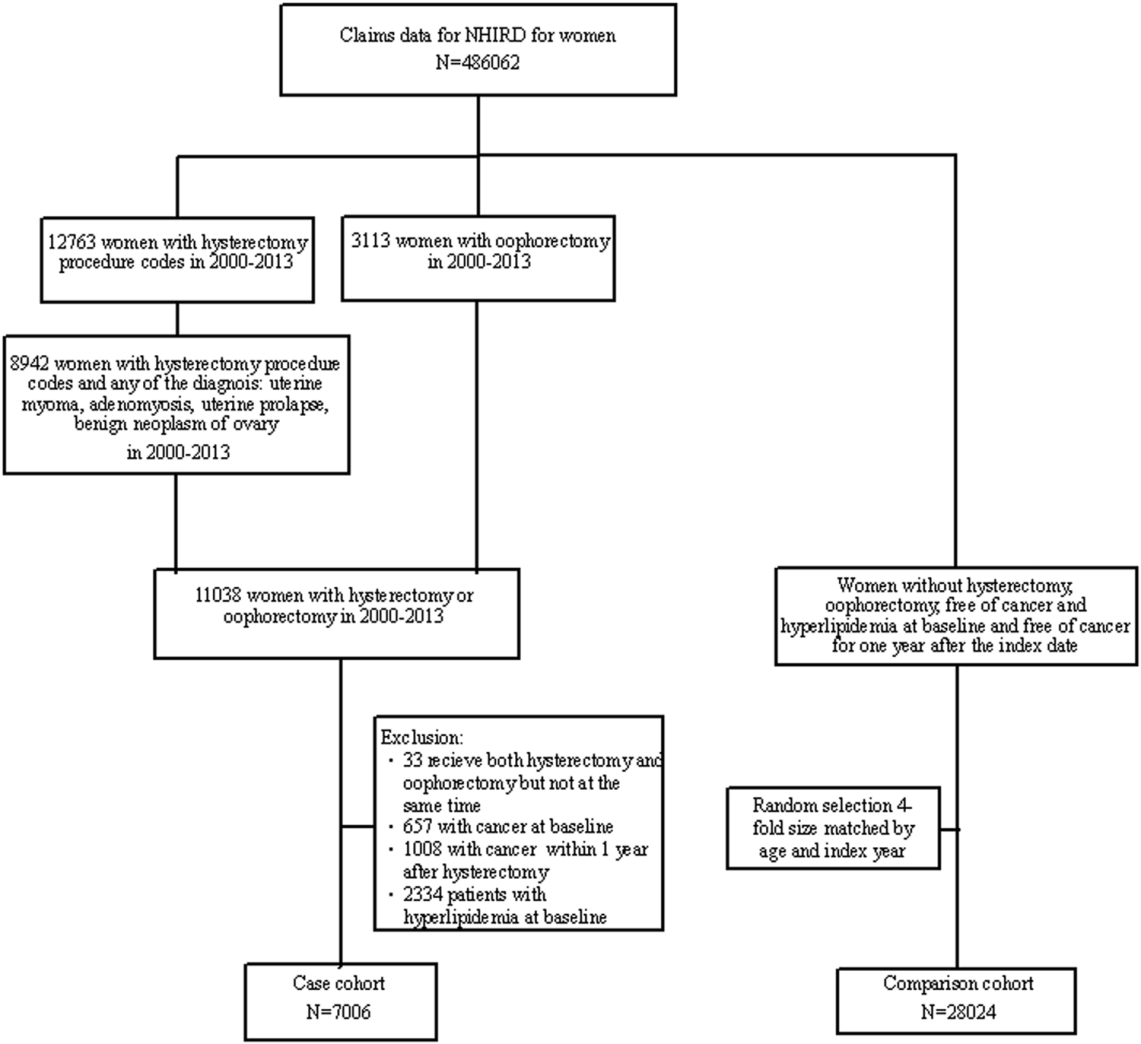
The flow chart for identifying study cohorts from the National Health Insurance Research Database.

We established a hysterectomy related case group, including women who had received only a hysterectomy, both a hysterectomy and an oophorectomy, and only an oophorectomy from 2000 to 2013. Cases were defined by the procedure codes of 65.5, 65.6, 68.3–5, 68.9^7^ and diagnoses codes of uterine myoma (ICD9 code: 218.9), adenomyosis (ICD9 code: 617.0), uterine prolapse (ICD9 code: 618), and ovarian tumor ICD9 code: 220. Women who had been diagnosed with hyperlipidemia (ICD9 code: 272) and/or cancer at baseline, or with cancer within 1 year after hysterectomy were excluded (Fig. 1). Similar exclusion criteria were applied for establishing the comparison group, which was randomly selected from women without the history of hysterectomy and oophorectomy, with a sample size 4-fold of the case group, frequency matched by the year of birth and the year of surgery. The validity of hysterectomy and hyperlipidemia in the claims data of NHIRD has been reported in previous reports^5–7^.

Women in both the hysterectomy case group and the comparison group were followed up until hyperlipidemia identified, death, withdrawal from the NHI program, or the end of 2013^11^.

Baseline comorbidities, including obesity (ICD9 codes: 278, 783.1, V77.8), diabetes (ICD9 code: 250), coronary arterial disease (CAD) (ICD9 code: 410–414), hypothyroidism (ICD9 code: 244), and hypertension (ICD9 codes: 401–405), were identified as related factors. We also included hormone therapy (ATC code: G03C, estrogens) before or after the baseline into account as a potential confounder. Women who received hormone therapy more than 30 days were considered as hormone therapy users.

### Statistical analysis

We applied Chi-square test to determine the differences of baseline characteristics for categorical variables and used Wilcoxon rank sum test to examined continuous variables between hysterectomy and comparison groups. Incidence of hyperlipidemia was estimated for both groups by the end of 2013. We used the Kaplan-Meier method to measure fractions free of hyperlipidemia during the follow-up period in two cohorts by the type of surgery, and used the Log-rank test to examine the difference. Incidence rates of hyperlipidemia, per 100 person-years, were calculated for women with only a hysterectomy, with both a hysterectomy and with only an oophorectomy, and for the comparison group, by age group (<45, 45–64 and ≥65 years), hormone therapy and comorbidities. Cox proportional hazards regression analysis was used to calculate each surgery group to the comparison group hazard ratio (HR) of hyperlipidemia and 95% confidence interval (CI). Adjusted hazard ratio (aHR) was estimated after controlling for covariates. All statistical analyses were performed using SAS software version 9.4 (SAS Institute INC., Carey, NC). A two-tailed p-value below 0.05 was considered as significant.

## Results

Table 1 shows that near a half of study population were less than 45 years old with a mean age of 46.9 years. The baseline comorbidities were more prevalent in the hysterectomy case groups than in the comparison group. The prevalence rate of hormone therapy was also higher in the hysterectomy case groups than in comparisons.

**Table 1:** Baseline characteristics in women with and without hysterectomy and oophorectomy.

	Comparison group	Case groups	Hysterectomy only	Hysterectomy + Oophorectomy	Oophorectomy only	p-value cases vs. controls
	(n = 28024)	(n = 7006)	(n = 5887)	(n = 563)	(n = 556)	
Age, years						
Mean (SD)	46.9 (9.01)	46.9 (9.00)	46.4 (8.34)	51.0 (8.35)	48.5 (13.8)	0.94
<45	13255 (47.3)	3396 (48.5)	3113 (52.9)	102 (18.1)	181 (32.6)	
45–64	13139 (46.9)	3200 (45.7)	2476 (42.1)	418 (74.3)	306 (55.0)	
≧65	1630 (5.82)	410 (5.85)	298 (5.06)	43 (7.64)	69 (12.4)	
Follow-up duration,						
Mean (SD), years	5.96 (2.84)	6.13 (2.63)	5.95 (3.95)	7.06 (4.05)	7.07 (4.36)	<0.0001
Hormone therapy*	3688 (13.2)	2177 (31.1)	1558 (26.5)	360 (63.9)	259 (46.6)	<0.0001
Comorbidity, n (%)						
Obesity	169 (0.60)	69 (0.98)	61 (1.04)	4 (0.71)	4 (0.72)	0.0005
Diabetes	1204 (4.30)	344 (4.91)	268 (4.55)	38 (6.75)	38 (6.83)	0.03
CAD	1592 (5.68)	480 (6.85)	365 (6.20)	53 (9.41)	62 (11.2)	0.0002
Hypothyroidism	238 (0.85)	79 (1.13)	75 (1.27)	2 (0.36)	2 (0.36)	0.03
Hypertension	3325 (11.9)	1086 (15.5)	819 (13.9)	127 (22.6)	140 (25.2)	<0.0001

^*^Hormone therapy for 30 days or longer; SD, standard deviation; CAD, coronary arterial disease.

**Figure 2:**
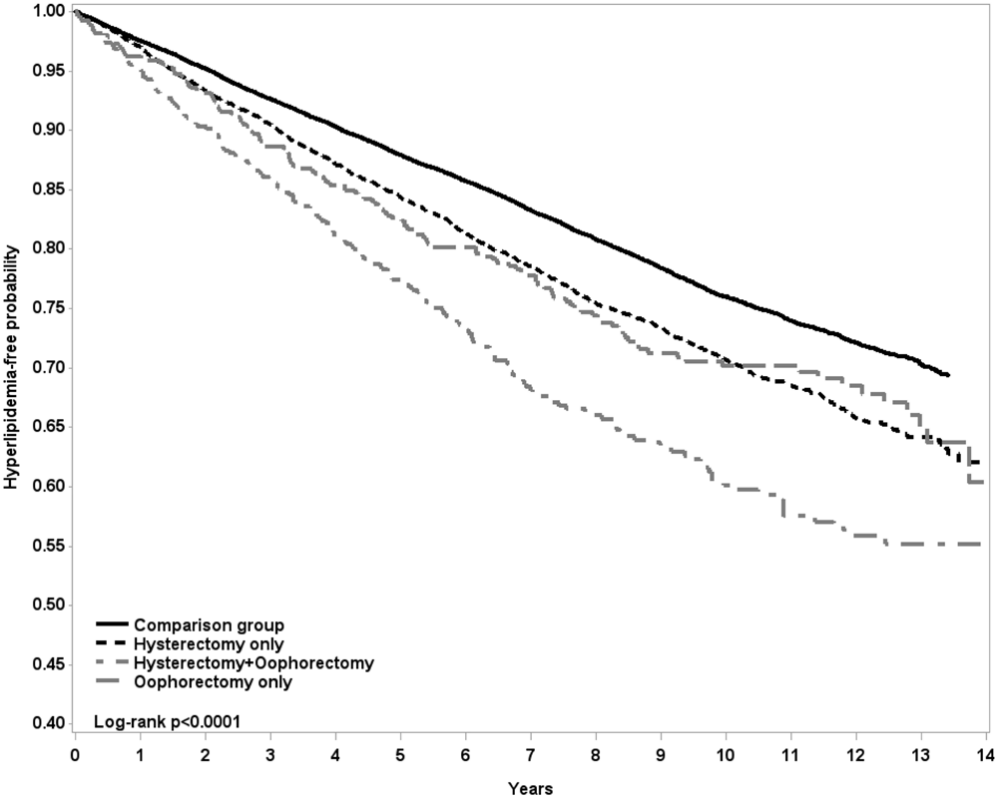
Kaplan-Meier curves of hyperlipidemia in women with only hysterectomy (black dashed line), with both hysterectomy and oophorectomy (gray dashed dot line), with only oophorectomy (gray dashed line) and in the comparison group (solid line) (Log-rank test p < 0.0001).

By the end of follow-up, the Kaplan-Meier analysis showed that the overall incident hyperlipidemia was 7.5% greater in the hysterectomy group than in the comparison group (p < 0.0001) (Fig. 2). The incidence density rate of hyperlipidemia was near 1.3 times greater in the hysterectomy group than in the comparison group (3.43 vs. 2.65 per 100 person-years), with an adjusted HR of 1.27 (95% CI = 1.19–1.35) for the hysterectomy group after controlling for age, oophorectomy, hormone therapy and comorbidities (Table 2). The hyperlipidemia incidence increased further for women had both hysterectomy and oophorectomy. The overall incident hyperlipidemia in women with oophorectomy alone was approximately similar to women with only hysterectomy. Those who had both a hysterectomy and an oophorectomy, and underwent hormone therapy, had an incidence of 5.40 per 100 person-years. The age stratified analysis showed that incidence rates of hyperlipidemia were higher in women aged 45–64 years than in youngers and the elderly. However, the age-specific surgery group to comparison group HRs were the highest in the young women for the three types of surgery. Obesity, hypertension and diabetes at the baseline were also associated with the elevated incidence of hyperlipidemia after the surgeries.

**Table 2:** Incidence of hyperlipidemia and hysterectomy groups to comparison group hazard ratio measured using Cox proportional hazards regression analysis.

	N	Event	PY	Rate^#^	Crude HR (95% CI)	Adjusted HR (95% CI)†
All						
Comparison group	28024	4708	177390	2.65	1 (reference)	1 (reference)
Hysterectomy only	5887	1202	35005	3.43	1.30 (1.22, 1.38)	1.27 (1.19, 1.35)
Hysterectomy + Oophorectomy	563	196	3976	4.93	1.85 (1.61, 2.14)	1.51 (1.31, 1.75)
Oophorectomy only	556	138	3928	3.51	1.32 (1.12, 1.56)	1.11 (0.93, 1.32)
**By age stratus**						
<45						
Comparison group	13255	1594	91505	1.74	1 (reference)	1 (reference)
Hysterectomy only	3113	552	20168	2.74	1.58 (1.43, 1.74)	1.49 (1.35, 1.65)
Hysterectomy + Oophorectomy	102	28	903	3.10	1.74 (1.19, 2.52)	1.71 (1.17, 2.50)
Oophorectomy only	181	24	1536	1.56	0.88 (0.59, 1.32)	1.37 (0.91, 2.06)
45–64						
Comparison group	13139	2770	76814	3.61	1 (reference)	1 (reference)
Hysterectomy only	2476	588	13170	4.46	1.24 (1.14, 1.36)	1.20 (1.10, 1.32)
Hysterectomy + Oophorectomy	418	153	2811	5.44	1.51 (1.28, 1.77)	1.34 (1.14, 1.59)
Oophorectomy only	306	97	1982	4.90	1.35 (1.11, 1.66)	1.13 (0.92, 1.39)
≥65						
Comparison group	1630	344	9071	3.79	1 (reference)	1 (reference)
Hysterectomy only	298	62	1667	3.72	0.98 (0.75, 1.29)	0.98 (0.74, 1.30)
Hysterectomy + Oophorectomy	43	15	262	5.72	1.49 (0.89, 2.50)	1.47 (0.87, 2.48)
Oophorectomy only	69	17	411	4.14	1.12 (0.69, 1.82)	0.99 (0.61, 1.63)
**Hormone therapy**						
No						
Comarison group	24336	3848	151874	2.53	1 (reference)	1 (reference)
Hysterectomy only	4329	814	24344	3.34	1.32 (1.23, 1.43)	1.34 (1.24, 1.45)
Hysterectomy + Oophorectomy	203	50	1270	3.94	1.55 (1.18, 2.05)	1.17 (0.89, 1.55)
Oophorectomy only	297	60	1836	3.27	1.29 (1.00, 1.67)	1.15 (0.89, 1.49)
Yes						
Comparison group	3688	860	25516	3.37	1 (reference)	1 (reference)
Hysterectomy only	1558	388	10661	3.64	1.08 (0.96, 1.22)	1.10 (0.98, 1.24)
Hysterectomy + Oophorectomy	360	146	2706	5.40	1.60 (1.34, 1.91)	1.60 (1.35, 1.91)
Oophorectomy only	259	78	2093	3.73	1.10 (0.87, 1.39)	1.05 (0.83, 1.32)
**Comorbidity**						
Obesity						
No	27855	4682	176624	2.65	1 (reference)	1 (reference)
Comparison group	5826	1185	34753	3.41	1.29 (1.21, 1.37)	1.26 (1.18, 1.35)
Hysterectomy only	559	195	3956	4.93	1.86 (1.61, 2.14)	1.51 (1.30, 1.75)
Hysterectomy + Oophorectomy	552	138	3910	3.53	1.33 (1.12, 1.57)	1.11 (0.94, 1.32)
Oophorectomy only						
Yes	169	26	766	3.4	1 (reference)	1 (reference)
Comparison group	61	17	252	6.75	1.96 (1.06, 3.61)	1.69 (0.89, 3.24)
Hysterectomy only	4	1	20	5.11	1.56 (0.21, 11.5)	1.70 (0.22, 13.5)
Hysterectomy + Oophorectomy	4	0	18	0	—	—
**Oophorectomy only**						
**Hypertension**						
No						
Comparison group	24699	3733	160185	2.33	1 (reference)	1 (reference)
Hysterectomy only	5068	956	31168	3.07	1.32 (1.23, 1.42)	1.31 (1.22, 1.41)
Hysterectomy + Oophorectomy	436	137	3241	4.23	1.80 (1.52, 2.13)	1.53 (1.28, 1.82)
Oophorectomy only	416	92	3179	2.89	1.23 (1.00, 1.51)	1.16 (0.94, 1.43)
Yes						
Comparison group	3325	975	17205	5.67	1 (reference)	1 (reference)
Hysterectomy only	819	246	3837	6.41	1.13 (0.98, 1.30)	1.09 (0.95, 1.26)
Hysterectomy + Oophorectomy	127	59	735	8.03	1.42 (1.10, 1.85)	1.40 (1.08, 1.83)
Oophorectomy only	140	46	750	6.14	1.09 (0.81, 1.47)	1.08 (0.80, 1.45)
**Diabetes**						
No						
Comparison group	26820	4355	171689	2.54	1 (reference)	1 (reference)
Hysterectomy only	5619	1115	33678	3.31	1.31 (1.23, 1.40)	1.28 (1.2, 1.37)
Hysterectomy + Oophorectomy	525	181	3778	4.79	1.88 (1.62, 2.19)	1.52 (1.31, 1.77)
Oophorectomy only	518	124	3712	3.34	1.31 (1.10, 1.57)	1.12 (0.94, 1.35)
Yes						
Comparison group	1204	353	5701	6.19	1 (reference)	1 (reference)
Hysterectomy only	268	87	1327	6.56	1.06 (0.84, 1.35)	1.05 (0.83, 1.33)
Hysterectomy + Oophorectomy	38	15	198	7.56	1.24 (0.74, 2.08)	1.13 (0.67, 1.92)
Oophorectomy only	38	14	216	6.48	1.10 (0.64, 1.87)	1.02 (0.59, 1.75)
CAD						
Comparison group Hysterectomy only	26432	4283	168788	2.54	1 (reference)	1 (reference)
Hysterectomy + Oophorectomy	5522	1102	33146	3.32	1.31 (1.23, 1.40)	1.28 (1.19, 1.37)
Oophorectomy only	510	177	3639	4.86	1.91 (1.64, 2.22)	1.55 (1.33, 1.81)
Yes	494	119	3546	3.36	1.32 (1.10, 1.58)	1.16 (0.97, 1.40)
Comparison group						
Hysterectomy only	1592	425	8602	4.94	1 (reference)	1 (reference)
Hysterectomy + Oophorectomy	365	100	1859	5.38	1.08 (0.87, 1.35)	1.07 (0.86, 1.33)
Oophorectomy only	53	19	337	5.63	1.15 (0.72, 1.82)	1.07 (0.68, 1.71)
Hypothyroidism	62	19	382	4.97	1.03 (0.65, 1.62)	0.94 (0.59, 1.50)
No						
Comparison group	27786	4665	176265	2.65	1 (reference)	1 (reference)
Hysterectomy only	5812	1189	34695	3.43	1.30 (1.22, 1.38)	1.27 (1.19, 1.35)
Hysterectomy + Oophorectomy	561	195	3963	4.92	1.86 (1.61, 2.14)	1.51 (1.31, 1.75)
Oophorectomy only	554	137	3927	3.49	1.32 (1.11, 1.56)	1.10 (0.93, 1.31)
Yes						
Comparison group	238	43	1125	3.82	1 (reference)	1 (reference)
Hysterectomy only	75	13	309	4.20	1.08 (0.58, 2.01)	1.09 (0.58, 2.06)
Hysterectomy + Oophorectomy	2	1	13	7.78	2.02 (0.28, 14.7)	1.43 (0.18, 11.1)
Oophorectomy only	2	1	2	55.2	14.5 (1.90, 111)	13.0 (1.66, 101)

^#^Per 100 person-years.

^†^Multivariable model adjusted for age, hormone therapy and comorbidities listed in Table 1.

CAD, coronary arterial disease; PY, person-years; HR, hazard ratio; CI, confidence interval.

## Discussion

Our study population encompassed mainly middle-aged women with a mean age of 46 years, which indicates that hysterectomy is a surgery prevalent in women in the middle ages instead of in the elderly. After a median follow-up time of 6.0 years, approximately one fifth of women with the hysterectomy developed hyperlipidemia. The risk of hyperlipidemia initiates in young women and reaches a peak in their 45–64 years.

Several cohort studies have reported the relationship between hysterectomy and risk of developing hyperlipidemia^8,12–14^. An Iran study found increased levels of triglyceride, total cholesterol and low density cholesterol in 31 women six months after their hysterectomy and bilateral salpingo-oophorectomy^14^. By comparing women with natural and surgical menopause, hysterectomy women may experience increased low-density lipoprotein and reduced high density cholesterol^12,13^. These findings are all fragmental and can not make a conclusion.

The Women’s Health Initiative Observational Study, a very large cohort study of white, black, Hispanic, and American Indian women in the USA, has shown that the increased cardiovascular disease risk in women with a hysterectomy is likely associated with baseline comorbidities^8^. In our study, the baseline comorbidities were more prevalent in the hysterectomy cohort than in the comparison cohort. The baseline hypertension and diabetes are associated with a further increase in hyperlipidemia, near 2-fold greater than women without these comorbidity.

After following-up 3,302 pre-menopausal women for 11 years, the Study of Women’s Health across the Nation revealed that levels of high density lipoprotein cholesterol, low density lipoprotein cholesterol and apolipoprotein B were similar for those with natural menopause and hysterectomy women with or without ovarian conservation over time^15^. The sample size of women with a hysterectomy (n = 183) in the Study may not be enough to observe significant difference^15^. The CARDIA Study followed up 1,045 young women ages 15–30 for 25 years, after their hysterectomy or natural menopause, and found a small increase in high-density lipoprotein cholesterol from their baseline values^16^. On the other hand, our study showed an increased incident hyperlipidemia in women post hysterectomy. The population size and ethnic characteristics of this nationwide cohort study in Taiwan were different from previous studies. Race-specific factors may have an impact on the lipid and lipoprotein changes characteristic of menopause and hormonal statuses^17^. In addition, our follow-up study included women of younger ages, when the decline in ovarian hormones would be less prominent. The incidence of hyperlipidemia in women of <45 years old was near 1.6 folds greater in those with a hysterectomy than in comparisons. The relative impact of hysterectomy on the hyperlipidemia risk is stronger in young women than in the elderly women.

Our study showed that hysterectomy alone or oophorectomy alone was associated with approximately 1.3-fold greater risk of developing hyperlipidemia than comparison women. Women with both hysterectomy and oophorectomy had an incidence of hyperlipidemia increased to 4.93 per 100 person-years, near 1.9-fold greater than comparison women. Age, hysterectomy alone, and the effect of bilateral oophorectomy are factors to be considered for the CVD risks. Hysterectomy without oophorectomy has been shown to interfere ovarian blood flow, resulting in premature ovarian failure and hormone-related effects on the vascular bed^18^. Alternatively, a bilateral oophorectomy can result in an abrupt fall in circulating estrogens and testosterone levels to cause menopause immediately^19^ and accelerate the increase in lipids and lipoproteins levels^20^.

Hysterectomy and/or oophorectomy can cause a surgical menopause. Following the menopausal transition, the estradiol level declines and testosterone progressively dominates the hormonal level, affecting the lipid metabolism^15^. Studies have also reported that metabolic conditions lead to the possible occurrence of the indications for hysterectomy and surgical menopause^15,16,21,22^. Adverse lipid levels tend to develop during and after menopause^23–25^. The Study of Women’s Health Across the Nation showed changes of lipid peaked in the late phase of menopause^25^. Women who have hysterectomy tend to reach physiological menopause earlier than women who do not have the surgery^26,27^. The average menopause age in Taiwan is 49.5 years^28^. The oophorectomy and comorbidity status may affect hormone and other disorders. A recent US cohort study with 113679 hysterectomy women found a greater hazard of mortality in those who had bilateral ovarian removed^29^. In our study, the hormone therapy status was adjusted in the data analysis.

### Strengths and Limitations of the Study

The novelty of this research lies in the population based assessment. This study composed of a large size of study population after a median follow-up period of 6 years containing all events of hysterectomy for benign diseases. This population based data could minimize selection bias. Our study also adjusts for some independent variables, such as diabetes and hypertension, known to influence the risk of CVD. Moreover, our data are representative for the general population and the comparison group is drawn from the same population by collected data from nationwide healthcare registers.

The potential limitation in the study is that hysterectomy women were more prevalent with comorbidities and thus were less healthy than general female population. In our baseline data, among 8942 women underwent hysterectomy, 1879 women (21.0%) had the history of hyperlipidemia and were excluded from the study. The baseline prevalence of hyperlipidemia was 1.27-fold greater in women with hysterectomy than in women without hysterectomy (16.6%) (data not shown). The baseline prevalence ratio was approximately similar to the hysterectomy cohort to the comparison cohort ratio of the newly developed incident hyperlipidemia (3.43 to 2.65 per 100 person-years, or 1.29). It is possible that hyperlipidemia appeared after the hysterectomy in this case would be a mere continuation of these underlying metabolic derangement rather than a result of the hysterectomy.

The other limitation was the potential misclassification in establishing study cohorts. We used hysterectomy procedure codes to identify the hysterectomy group. However, our further data analysis revealed that approximately 30% of women identified from the procedure codes lack of the diagnosis code. These women were thus mistakenly excluded from this study. Otherwise, this study should be highly reliable because the claims data have been peer reviewed by specialists. Women living in Taiwan are relatively homogenous, which may increase the internal validity and reliability to the findings. But, the ability to generalize our findings to other population is unknown. We were also unable to trace the surgical history before 1996.

## Conclusion

We concluded that women with a hysterectomy are at an elevated risk of hyperlipidemia compared to women of the same age without the surgery. The risk is increased further for those who have both a hysterectomy and an oophorectomy. Hysterectomy is indicated for a variety of benign disorders, providing reproductive and perimenopausal women with a definitive surgical solution to gynecological disorders. Women with these surgeries should be aware of the potential risk of developing hyperlipidemia. Oophorectomy could play another important role and future research that focuses on the oophorectomy status should be conducted.

## Data Availability

The data that support the findings of this study are disclosed in the paper. The raw data should be requested from the National Health Insurance Department.
